# Knowledge, attitude, confidence, and educational needs of palliative care in nurses caring for non-cancer patients: a cross-sectional, descriptive study

**DOI:** 10.1186/s12904-020-00581-6

**Published:** 2020-07-11

**Authors:** Sanghee Kim, Kyunghwa Lee, Sookyung Kim

**Affiliations:** 1 Mo-Im Kim Nursing Research Institute, Yonsei University College of Nursing, 50-1 Yonsei-ro, Seodaemun-gu, Seoul, 03722 Republic of Korea; 2 Konyang University College of Nursing, 158, Gwanjeodong-ro, Seo-gu, Daejeon, 35365 Republic of Korea; 3 Department of Nursing, Yonsei University Graduate School, 50-1 Yonsei-ro, Seodaemun-gu, Seoul, 03722 Republic of Korea

**Keywords:** Confidence, Education, Needs, Nurses, Palliative care

## Abstract

**Background:**

Palliative care is a patient-centred, integrated approach for improving quality of life for both patients facing life-threatening illnesses and for their families. Although there has been increased interest in palliative care for non-cancer patients, the palliative care competency of nurses who care for non-cancer patients has rarely been investigated. This study described the palliative care knowledge, attitude, confidence, and educational needs in nurses who care for patients with congestive heart failure, stroke, end-stage renal disease, and end-stage liver disease; explored the relationships between those variables; and identified factors affecting nurses’ palliative care confidence.

**Methods:**

A cross-sectional, descriptive, correlational design was employed; data collection was conducted at a tertiary hospital in Seoul, Korea. Nurses who were working in general wards and intensive care units (*N* = 102) completed valid and reliable self-administered questionnaires. Descriptive statistics, frequencies, independent *t*-tests, one-way ANOVA, Pearson’s correlations, and multiple regression were conducted to analyse the data.

**Results:**

Nurses’ palliative care knowledge level was low (9.73 ± 2.10; range = 0–20) and their attitude toward palliative care was moderate (87.97 ± 6.93, range: 30–120). Knowledge was significantly correlated with attitude (*r* = .29, *p* = .003). Nurses were highly confident in pain and symptom management but demonstrated high educational needs for managing human and material resources to provide palliative care. Previous training in hospice, palliative, and EOL care was a significant and modifiable factor that affected nurses’ confidence (std. *β* = 0.25, *p* = .010).

**Conclusions:**

To facilitate high-quality palliative care for non-cancer patients and families, nursing education programs should be developed to address nurses’ knowledge level, confidence level, and educational needs. This study provides relevant information that can be utilised to develop palliative care educational programs for nurses who care for non-cancer patients.

## Background

Palliative care includes patient-centred physical, psychosocial, and spiritual care to improve quality of life for both patients facing life-threatening illnesses and for their family members [1]. Palliative care has been proposed as a continuous care model beginning at disease diagnosis and continuing through the patients’ end-of-life (EOL) [2]. The need for palliative care is increasing worldwide because of population aging, an increased prevalence of chronic diseases, and increased interest in quality of life [3–5]. For example, based on an analysis of mortality statistics in England and Wales from 2006 to 2014, 160,000 more people annually will need palliative care by 2040, if the current mortality trend continues [6]. By 2060, an estimated 48 million people will have died with serious health-related suffering. In addition, the global burden of serious health-related suffering will rapidly increase in low-income countries and among older people [4]. Further, although hospital deaths have consistently continued to increase [7, 8], information related to patients’ and families’ experience of palliative care in a hospital setting is still limited [9].

As the need for palliative care became more apparent, the range of palliative care delivery broadened beyond cancer patients to include non-cancer patients, such as those with congestive heart failure (CHF), stroke, renal failure, spinal muscular atrophy, and chronic obstructive pulmonary disease [10–13]. It has been suggested that palliative care patterns should differ for cancer patients versus non-cancer patients because of distinct disease progression (e.g., steady progression and clear terminal phase for cancer versus gradual decline punctuated by episodes of acute deterioration and more sudden death for respiratory or heart failure [14]. Although palliative care is beneficial for non-cancer patients and their families, the use of palliative care services among non-cancer patients is much lower than for cancer patients, and palliative care referral tends to be later [15–18]. According to the Clinical Practice Research Datalink in England, in 2009, only 234 (7%) of 3122 patients with heart failure were given palliative care by family physicians or referred to special palliative care services, compared to 3669 (48%) of 7608 cancer patients [15]. Also, one study showed that patients with cardiovascular disease had very advanced disease when referred to palliative care [18].

To provide effective and high quality palliative care, it is necessary to integrate knowledge, skills, and favourable attitudes toward palliative care [19, 20]. Nurses who are knowledgeable, skilful, and comfortable providing EOL care could improve the quality of life and satisfaction of patients and their families in hospital settings [21]. For this reason, in the United States, ‘train-the-trainer’ education programs, such as Education on Palliative and End of Life Care and End of Life Nursing Education Consortium (ELNEC), have provided essential EOL care information to doctors and nurses working in clinical settings [22–24]. Also, in Korea the ELNEC-Core course has been implemented since 2009 and palliative care education programs have been extended to geriatric and paediatric areas [25]. However, compared to the high level of awareness among nursing professionals providing palliative care for cancer patients, efforts to improve palliative care for non-cancer patients are insufficient. Previous studies reported that nurses working in oncology units or cancer centres showed higher levels of palliative care knowledge than nurses working in general wards or intensive care units (ICUs) [26, 27]. Hence, it is necessary to assess the palliative care knowledge levels and attitudes, develop and implement guidelines, and design practical training programs to produce skilled nursing professionals who can provide palliative care for non-cancer patients.

The health care professionals’ lack of confidence in provide palliative care decreases quality of care for hospitalized patients [28]. Previous studies mentioned that education is fundamental for improving health care professionals’ palliative care confidence [29]. For example, the European Certificate in Essential Palliative Care, an eight-week home study course, effectively improved health care professionals’ confidence in palliative care, such as symptom management, communication, and applying spiritual approaches in clinical settings [29]. Also, health care professionals reported a high level of palliative care educational needs [30]. Palliative care confidence levels and educational needs should be investigated to develop evidence-based education programs.

This study examined the knowledge levels, attitudes, confidence, and educational needs of palliative care in nurses caring for non-cancer patients and identify factors affecting nurses’ confidence.

## Methods

### Study aims

The study aimed to (1) examine palliative care knowledge, attitudes, confidence, and educational needs in nurses who care for patients with CHF, stroke, end-stage renal disease (ESRD), and end-stage liver disease (ESLD); (2) explore the relationships between nurses’ palliative care knowledge, attitudes, confidence, and educational needs; and (3) identify factors affecting nurses’ confidence in providing palliative care.

### Study design

A cross-sectional, descriptive, correlational design was used for this study.

### Sample and setting

Convenience sampling was used to recruit participants from a tertiary hospital in Seoul, Korea that employed more than 2500 nurses. The inclusion criteria were: (1) nurses working in general wards and ICUs where patients with CHF, stroke, ESRD, or ESLD were hospitalised; (2) worked as a nurse for at least 1 year; and (3) able to understand the study purpose and voluntarily provide written, informed consent.

**Fig. 1:**
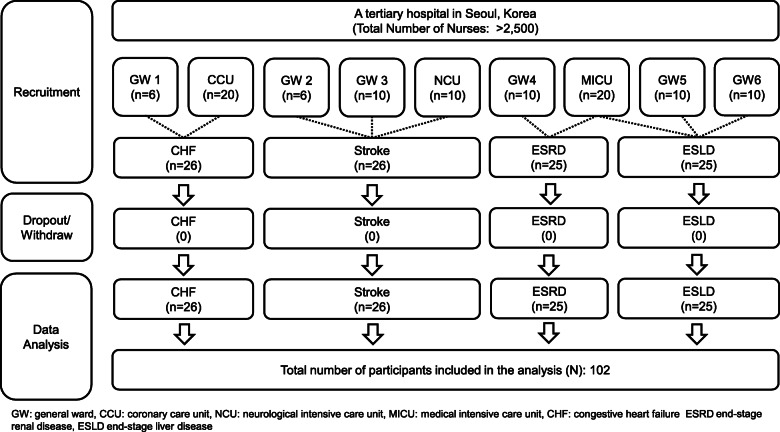
Participant recruitment flow chart

The sample size was calculated for correlation analyses, a medium effect size (Cohen’s *ρ* = 0.3), a two-tailed significance level of .05, and power of 0.85 using G*power version 3.1.9.2 [31]. Ninety-three participants were required based on these assumptions. Projecting a 10% drop-out rate, the required sample size was 102 participants. One hundred two nurses who worked in six general wards and three ICUs participated. There were no dropouts or withdrawals (Fig. 1).

### Data collection

Data were collected from August 18 to September 4, 2018. With the cooperation of unit managers and department of nursing units, researchers visited the general wards and ICUs and explained the study purpose and methods to potential participants. The researchers also explained that the participants were free to withdraw at any time. Participants who voluntarily provided written consent to participate then completed self-administered questionnaires in paper form after their shift. After all participants completed the questionnaires, the researchers revisited the wards and ICUs and collected the completed questionnaires.

### Instruments

#### General characteristics

Based on previous studies of factors associated with nurses’ palliative care knowledge, attitude, needs, and confidence [25, 32], we assessed nurses’ general characteristics using a questionnaire that asked about age, sex, marital status, educational background, religious affiliation, main type of patients cared for (i.e., disease type), work environment (i.e., general ward or ICU), current (in present working department) and total clinical experience, position, whether they had obtained a nurse practitioner certificate, and whether they had received hospice, palliative, or EOL care education.

#### Knowledge

Knowledge of palliative care was measured with the Palliative Care Quiz for Nursing (PCQN), developed by Ross, McDonald, and McGuinness [33], and translated into Korean by Kim and colleagues [34]. The instrument comprises 20 items about philosophy and principles (4 items), pain and symptom management (13 items), and psychosocial aspects of care (4 items). Total scores range from 0 to 20, with higher scores indicating higher knowledge levels (1 point for correct responses; 0 points for incorrect or ‘*I do not know*’ responses). The systemic content validity index was reported as 0.85 for the Korean version of the PCQN [34].

#### Attitude

Attitude toward palliative care was measured with the Frommelt Attitude Toward Care of the Dying Scale (Form A for nurses), originally developed by Frommelt [35] and translated into Korean by Cho and Kim [36]. The original instrument consisted of 30 items measured on a five-point Likert scale; however, the Korean version employs a four-point Likert scale (1 = *strongly disagree* to 4 = *strongly agree*) to prevent central convergence. Total scores range from 30 to 120, with higher scores indicating a more positive attitude toward caring for terminally ill patients and their family members. The content validity index of the original instrument was 1.0 and interrater agreement was 0.98 [35]. The Pearson correlation coefficient was 0.90 to 0.94 in the original instrument [35], and Cronbach’s alphas were 0.86 in the Korean version [36] and 0.78 in this study.

#### Confidence and educational needs

Palliative care confidence and educational needs were measured with the Self-reporting Confidence and Educational Needs in Hospice Care instrument, developed by Kwon and colleagues [37], which includes 22 items measured on a four-point Likert scale, and has four subdomains: (1) pain and symptom management (7 items) includes symptom assessment and nursing care for common symptoms; (2) counselling (5 items) includes risk management, communication, and spiritual care; (3) management (5 items) includes managing human and material resources related to palliative care; and (4) program (5 items) includes systematic and ethical approaches to protect patients’ and families’ rights in palliative care (i.e., decision-making, care planning, bereavement management etc.). Scores range from 1 to 4 (1 = *I have not learned at all/I do not need at all* to 4 = *I know it well and I can do it proficiently/it is very necessary*), with higher scores indicating higher levels of palliative care confidence or educational needs. In the original study, Cronbach’s alphas for the confidence and educational needs subdomains ranged from 0.82 to 0.92 and 0.87 to 0.92, respectively [37]. In this study, Cronbach’s alphas ranged from 0.85 to 0.96 and 0.92 to 0.97, respectively.

### Data analyses

Data were analysed with IBM SPSS Statistics, version 23.0 for Windows (IBM Corp., Armonk, NY, USA). Descriptive statistics and frequencies showed the distributions of participant characteristics, knowledge, attitude, confidence, and educational needs. Independent *t*-tests and one-way ANOVA were used to compare the four main variables by general characteristics. Pearson’s correlations were used to examine relationships between the four main variables. Finally, multiple regression was used to identify factors that affected nurses’ confidence in providing palliative care.

## Results

### Participant characteristics

Participant characteristics are shown in Table 1. Ninety-eight participants (96.1%) were women; mean age 32.4 ± 7.1 (range: 23–52); 16 participants (15.7%) had a Master’s degree. For main type of patients (i.e., CHF, stroke, ESRD, or ESLD), 25 to 26 participants were included from each disease type. Fifty-five participants (53.9%) worked in ICUs. Participants’ mean total clinical experience after nursing school graduation was 107.3 ± 86.2 months, and mean current work setting clinical experience was 67.6 ± 50.9 months. Eighty participants (78.4%) were staff nurses. Forty-three participants reported education post nursing school graduation in hospice, palliative, or EOL care, with most courses (83.3%) having a length of eight or fewer hours.

**Table 1:** Participants’ Characteristics (*N* = 102)

Characteristic	*n* (%)	Mean ± *SD*	Median (range)
Sex			
Women	98 (96.1)		
Men	4 (3.9)		
Age (years)		32.4 ± 7.1	30.0 (23.0–52.0)
Marital status			
Unmarried	61 (59.8)		
Married	40 (39.2)		
Other	1 (1)		
Educational background			
Associate (College)	9 (8.8)		
Bachelors (University)	77 (75.5)		
Masters	16 (15.7)		
Religious affiliation			
None	55 (53.9)		
Catholic	16 (15.7)		
Protestant	29 (28.4)		
Buddhism	2 (2.0)		
Main disease group			
CHF	26 (25.5)		
Stroke	26 (25.5)		
ESRD	25 (24.5)		
ESLD	25 (24.5)		
Work environment			
General ward	47 (46.1)		
ICU	55 (53.9)		
Clinical experience (months)			
Current (in present working department)		67.6 ± 50.9	50.0 (3–240)
Total^†^		107.3 ± 86.2	65.5 (12–375)
Position			
Staff nurse	80 (78.4)		
Charge nurse	19 (18.6)		
Head nurse	3 (2.9)		
Certificate of nurse practitioner			
Yes	9 (8.8)		
No	93 (91.2)		
Received hospice, palliative, and EOL care education^‡^			
Yes	43 (42.2)		
≤ 8 h	35 (83.3)		
9–16 h	7 (16.7)		
No	58 (56.9)		

*SD* standard deviation, *CHF* congestive heart failure, *ESRD* end-stage renal disease, *ESLD* end-stage liver disease, *ICU* intensive care unit, *EOL* end-of-life

^†^*n* = 100, ^‡^*n* = 101

### Knowledge

Table 2 shows participant knowledge levels and the correct and incorrect answer rates per item. The mean total knowledge score was 9.73 ± 2.10 (range 0–20); the philosophy and principle mean score was lower (1.58 ± 0.83, range: 0–4) than the other three subdomains. Placebo use in pain treatment (item no. 13) and inevitable burnout of palliative care workers (item no. 17) had the highest number of incorrect responses. Psychosocial support for family members (item no. 5) and adjuvant therapies for pain management (item no. 4) were answered correctly by nearly all participants.

**Table 2:** Levels of Knowledge and Correct-Incorrect Answer Rates per Item (*N* = 102)

Knowledge (Range)		Total (*N* = 102)	CHF (*n* = 26) (a)	Stroke (*n* = 26) (b)	ESRD (*n* = 25) (c)	ESLD (*n* = 25) (d)		*F(p)*
		Mean ± *SD*	Mean ± *SD*	Mean ± *SD*	Mean ± *SD*	Mean ± *SD*		
Total (0–20)		9.73 ± 2.10	9.35 ± 1.74	9.27 ± 2.32	9.52 ± 2.33	10.80 ± 1.66		3.13 (.029) (b < d)
Philosophy and principle (0–4)		1.58 ± 0.83	1.62 ± 0.90	1.23 ± 0.77	1.72 ± 0.79	1.76 ± 0.78		2.29 (.084)
Pain and symptom management (0–13)		6.39 ± 1.65	5.96 ± 1.54	6.27 ± 1.78	6.04 ± 1.65	7.32 ± 1.31		3.99 (.010) (a, c < d)
Psychosocial aspects of care (0–3)		1.75 ± 0.84	1.77 ± 0.86	1.77 ± 0.71	1.76 ± 0.93	1.72 ± 0.89		0.02 (.996)
Item No	Subdomain	Item (answer)		Response (%)				
				CHF (*n* = 26)	Stroke (*n* = 26)	ESRD (*n* = 25)	ESLD (*n* = 25)	Total (*N* = 102)
				Correct (incorrect)	Correct (incorrect)	Correct (incorrect)	Correct (incorrect)	Correct (incorrect)
1	Philosophy and principle	Palliative care is appropriate only in situations where there is evidence of a downhill trajectory or deterioration (F)		18.6 (6.9)	18.6 (6.9)	17.6 (6.9)	19.6 (4.9)	74.5 (25.5)
9	Philosophy and principle	The provision of palliative care requires emotional detachment (F)		5.9 (19.6)	3.9 (21.6)	7.8 (16.7)	8.8 (15.7)	26.5 (73.5)
12	Philosophy and principle	The philosophy of palliative care is compatible with that of aggressive treatment (T)		12.7 (12.7)	8.8 (16.7)	14.7 (9.8)	14.7 (9.8)	51.0 (49.0)
17	Philosophy and principle	The accumulation of losses renders burnout inevitable for those who seek work in palliative care (F)		3.9 (21.6)	0.0 (25.5)	2.0 (22.5)	0.0 (24.5)	5.9 (94.1)
2	Pain and symptom management	Morphine is the standard used to compare the analgesic effect of other opioids (T)		3.9 (21.6)	7.8 (17.6)	8.8 (15.7)	10.8 (13.7)	31.4 (68.6)
3	Pain and symptom management	The extent of the disease determines the method of pain treatment (F)		9.8 (15.7)	10.8 (14.7)	10.8 (13.7)	6.9 (17.6)	38.2 (61.8)
4	Pain and symptom management	Adjuvant therapies are important in managing pain (T)		25.5 (0.0)	24.5 (1.0)	21.6 (2.9)	24.5 (0.0)	96.1 (3.9)
6	Pain and symptom management	During the last days of life, the drowsiness associated with electrolyte imbalance may decrease the need for sedation (T)		2.0 (23.5)	4.9 (20.6)	2.9 (21.6)	2.9 (21.6)	12.7 (87.3)
7	Pain and symptom management	Drug addiction is a major problem when morphine is used on a long-term basis for the management of pain (F)		5.9 (19.6)	9.8 (15.7)	7.8 (16.7)	9.8 (14.7)	33.3 (66.7)
8	Pain and symptom management	Individuals who are taking opioids should also follow a bowel regimen (T)		22.5 (2.9)	21.6 (3.9)	20.6 (3.9)	23.5 (1.0)	88.2 (11.8)
10	Pain and symptom management	During the terminal stages of an illness, drugs that can cause respiratory depression are appropriate for the treatment of severe dyspnoea (T)		4.9 (20.6)	8.8 (16.7)	3.9 (20.6)	9.8 (14.7)	27.5 (72.5)
13	Pain and symptom management	The use of placebos is appropriate in the treatment of some types of pain (F)		2.0 (23.5)	1.0 (24.5)	2.9 (21.6)	0.0 (24.5)	5.9 (94.1)
14	Pain and symptom management	In high doses, codeine causes more nausea and vomiting than morphine (T)		9.8 (15.7)	6.9 (18.6)	6.9 (17.6)	13.7 (10.8)	37.3 (62.7)
15	Pain and symptom management	Suffering and physical pain are synonymous (F)		18.6 (6.9)	14.7 (10.8)	17.6 (6.9)	18.6 (5.9)	69.6 (30.4)
16	Pain and symptom management	Demerol is not an effective analgesic in the control of chronic pain (T)		10.8 (14.7)	16.7 (8.8)	14.7 (9.8)	18.6 (5.9)	60.8 (39.2)
18	Pain and symptom management	Manifestations of chronic pain are different from those of acute pain (T)		19.6 (5.9)	18.6 (6.9)	14.7 (9.8)	20.6 (3.9)	73.5 (26.5)
20	Pain and symptom management	The pain threshold is lowered by anxiety or fatigue (T)		16.7 (8.8)	13.7 (11.8)	14.7 (9.8)	19.6 (4.9)	64.7 (35.3)
5	Psychosocial and spiritual care	It is crucial for family members to remain at the bedside until death occurs (T)		24.5 (1.0)	24.5 (1.0)	23.5 (1.0)	24.5 (0.0)	97.1 (2.9)
11	Psychosocial and spiritual care	Men generally reconcile their grief more quickly than women (F)		12.7 (12.7)	6.9 (18.6)	10.8 (13.7)	9.8 (14.7)	40.2 (59.8)
19	Psychosocial and spiritual care	The loss of a distant or contentious relationship is easier to resolve than the loss of one who is close or intimate (F)		7.8 (17.6)	13.7 (11.8)	8.8 (15.7)	7.8 (16.7)	38.2 (61.8)

*SD* standard deviation, *CHF* Congestive heart failure, *ESRD* end-stage renal disease, *ESLD* end-stage liver disease, Post Hoc: Tukey HSD

The total knowledge score was significantly higher among participants working in general wards (10.36 ± 2.21) than ICUs (9.18 ± 1.86) (*t* = 2.93, *p* = .004). Also, the total knowledge score was significantly associated with the main disease group (*F* = 3.13, *p* = .029); participants who cared for patients with ESLD had significantly higher knowledge scores than participants who cared for patients with stroke. Among three subdomains, the mean pain and symptom management score was significantly associated with the main disease group (*F* = 3*.99, p* = .010); participants who cared for ESLD patients had significantly higher knowledge scores than participants who cared for CHF and ESRD patients.

### Attitude

Table 3 shows participants’ attitude level. The total attitude score was 87.97 ± 6.93 (range: 30–120). Attitude was not significantly associated with work environment, but was significantly associated with main disease group (*F* = 2.87, *p* = .040); participants who cared for ESLD patients had significantly higher attitude scores than participants who cared for stroke patients. Communication with the dying person about impending death (item no. 3) and patients’ emotional expression at the end of life (item no 26) had the lowest mean scores.

**Table 3:** Levels of Attitude (*N* = 102)

Attitude (30–120)	Total (*N* = 102)	CHF (*n* = 26) (a)	Stroke (*n* = 26) (b)	ESRD (*n* = 25) (c)	ESLD (*n* = 25) (d)	*F(p)*
	Mean ± *SD*	Mean ± *SD*	Mean ± *SD*	Mean ± *SD*	Mean ± *SD*	
	87.97 ± 6.93	87.08 ± 5.54	86.19 ± 5.98	87.40 ± 8.65	91.32 ± 6.47	2.87 (.040) (b < d)
Item No	Item (range: 1–4)					Mean ± *SD*
1	Giving nursing care to the dying person is a worthwhile learning experience.					3.14 ± 0.53
2	Death is not the worst thing that can happen to a person.					2.36 ± 0.78
3^†^	I would be uncomfortable talking about impending death with the dying person.					1.72 ± 0.57
4	Nursing care for the patient’s family should continue throughout the period of grief and bereavement.					3.38 ± 0.55
5^†^	I would not want to be assigned to care for a dying person.					2.39 ± 0.80
6^†^	The nurse should not be the one to talk about death with the dying person.					3.05 ± 0.71
7^†^	The length of time required to give nursing care to a dying person would frustrate me.					2.40 ± 0.69
8^†^	I would be upset when the dying person I was caring for gave up hope of getting better.					2.66 ± 0.65
9^†^	It is difficult to form a close relationship with the family of the dying person.					2.75 ± 0.64
10	There are times when death is welcomed by the dying person.					2.77 ± 0.51
11^†^	When a patient asks, “Nurse am I dying?,” I think it is best to change the subject to something cheerful.					3.23 ± 0.58
12	The family should be involved in the physical care of the dying person.					3.09 ± 0.65
13^†^	I would hope the person I’m caring for dies when I am not present.					2.59 ± 0.68
14^†^	I am afraid to become friends with a dying person.					2.71 ± 0.68
15^†^	I would feel like running away when the person actually died.					3.20 ± 0.75
16	Families need emotional support to accept the behavior changes of the dying person.					3.58 ± 0.55
17^†^	As a patient nears death, the nurse should withdraw from his/her involvement with the patient.					2.81 ± 0.67
18	Families should be concerned about helping their dying member make the best of his/her remaining life.					3.37 ± 0.54
19^†^	The dying person should not be allowed to make decisions about his/her physical care.					3.60 ± 0.57
20	Families should maintain as normal an environment as possible for their dying member.					3.25 ± 0.59
21^†^	It is beneficial for the dying person to verbalize his/her feelings.					3.25 ± 0.59
22	Nursing care should extend to the family of the dying person.					3.48 ± 0.58
23	Nurses should permit dying persons to have flexible visiting schedules.					3.47 ± 0.52
24	The dying person and his/her family should be the in-charge decision makers.					3.39 ± 0.57
25	Addiction to pain relieving medication should not be a concern when dealing with a dying person.					2.80 ± 0.83
26^†^	I would be uncomfortable if I entered the room of a terminally ill person and found him/ her crying.					1.85 ± 0.53
27	Dying persons should be given honest answers about their condition.					3.07 ± 0.62
28^†^	Educating families about death and dying is not a nursing responsibility.					3.02 ± 0.78
29^†^	Family members who stay close to a dying person often interfere with the professionals job with the patient.					2.47 ± 0.59
30	It is possible for nurses to help patients prepare for death.					3.11 ± 0.49

*SD* standard deviation, *CHF* Congestive heart failure, *ESRD* end-stage renal disease, *ESLD* end-stage liver disease, Post Hoc: Tukey HSD ^†^reverse coding

### Confidence and educational needs

Participants’ confidence and educational needs are shown in Table 4. Participants were highly confident about ‘pain and symptom management’ (3.16 ± 0.59), but not confident about ‘management’ (2.24 ± 0.78), which was similarly reflected in the educational needs scores for these areas. Participants responded that they were least confident about ‘volunteer management’ and had the highest educational need for ‘stress management for employees’. Participants who had received hospice, palliative, or EOL care education showed a significantly higher confidence level for ‘pain and symptom management’ and ‘management’ than did participants without such education (*t* = − 3.16, *p* = .002; *t* = − 2.61, *p* = .010). Participants caring for ESLD patients showed higher confidence levels for ‘pain and symptom management’, ‘management’, and ‘program’ than the other participants (*F* = 6.88, *p* = <.001; *F* = 3.41, *p* = .021; *F* = 5.11, *p* = .003); accordingly, they also showed the lowest educational need for ‘pain and symptom management’ and ‘program’ among all participants (*F* = 3.29, *p* = .024; *F* = 2.84, *p* = .042).

**Table 4:** Confidence Levels, and Educational Needs (*N* = 102)

Category		Theme							Confidence		Educational needs
									Mean ± *SD*		Mean ± *SD*
Pain and symptom management (1–4)		Pain and symptom assessment							3.43 ± 0.76		2.87 ± 0.69^‡^
		Pain management (pharmacological, non-pharmacological)							3.34 ± 0.74		2.99 ± 0.63^‡^
		Symptom management (confusion, delirium, dyspnoea, sleep disorder, nausea/vomiting, ascites)							3.32 ± 0.71		3.02 ± 0.64^‡^
		Nutrition and excretion management							3.13 ± 0.90		2.89 ± 0.67^‡^
		Lymphedema management							2.60 ± 0.98		2.98 ± 0.64^‡^
		History taking							3.14 ± 0.83		2.89 ± 0.65^‡^
		EOL care							3.17 ± 0.73		3.05 ± 0.63^‡^
		Total							3.16 ± 0.59		2.96 ± 0.53
Counselling (1–4)		Crisis management for individuals and families							2.80 ± 0.82		2.99 ± 0.64^‡^
		Communication and counselling							2.91 ± 0.85		3.00 ± 0.64^§^
		Spiritual needs assessment							2.60 ± 0.86		2.95 ± 0.65^§^
		Spiritual care							2.55 ± 0.87		2.95 ± 0.63^§^
		Understanding and care for the psychological shock of a patient at EOL and patient’s family members							2.67 ± 0.90		3.04 ± 0.62^§^
		Total							2.71 ± 0.79		2.99 ± 0.59
Management (1–4)		Stress management for employees							2.71 ± 0.84		3.09 ± 0.60^‡^
		Management and role of hospice team							2.26 ± 0.88		3.06 ± 0.57^‡^
		Quality improvement of hospice							2.26 ± 0.90		3.08 ± 0.61^‡^
		Hospice management (operating system of human resource/facilities/financial management)							2.04 ± 0.90		2.98 ± 0.65^‡^
		Volunteer management							1.92 ± 0.92		2.92 ± 0.61^‡^
		Total							2.24 ± 0.78		3.03 ± 0.54
Program (1–4)		Truth notification and protecting patients’ rights							2.38 ± 0.91^‡^		2.99 ± 0.60^§^
		Complementary alternative therapy to improve comfort							2.37 ± 0.89^†^		3.03 ± 0.58^§^
		Education and support for families							2.72 ± 0.87		2.95 ± 0.63^§^
		Bereavement management for families: types, methods, and care management for bereaved families							2.45 ± 0.92^†^		2.95 ± 0.61^§^
		Ethical decision-making and care planning							2.48 ± 0.87^†^		3.01 ± 0.58^§^
		Total							2.48 ± 0.79		2.99 ± 0.56
Characteristic	*n* (%)	Total Confidence		Confidence 1		Confidence 2		Confidence 3		Confidence 4	
		Mean ± *SD*	*t/F(p)*	Mean ± *SD*	*t/F(p)*	Mean ± *SD*	*t/F(p)*	Mean ± *SD*	*t/F(p)*	Mean ± *SD*	*t/F(p)*
*Received hospice, palliative, and EOL care education*^†^											
Yes	43 (42.6)	2.86 ± 0.55	−2.51 (.014)	3.36 ± 0.48	−3.16 (.002)	2.83 ± 0.76	−1.40 (.164)	2.46 ± 0.75	−2.61 (.010)	2.57 ± 0.77	− 1.16 (.250)
No	58 (57.4)	2.56 ± 0.62		3.00 ± 0.62		2.61 ± 0.81		2.06 ± 0.77		2.38 ± 0.80	
*Main disease group cared for*											
CHF (a)	26 (25.5)	2.62 ± 0.52	5.82 (.001) (a, b, c < d)	3.06 ± 0.46	6.88 (< .001) (a, b < d)	2.71 ± 0.84	1.96 (.126)	2.10 ± 0.81	3.41 (.021) (a, b < d)	2.44 ± 0.73	5.11 (.003) (b, c < d)
Stroke(b)	26 (25.5)	2.44 ± 0.72		2.86 ± 0.70		2.45 ± 0.90		2.07 ± 0.90		2.19 ± 0.87	
ESRD(c)	25 (24.5)	2.64 ± 0.55		3.20 ± 0.55		2.69 ± 0.71		2.14 ± 0.65		2.32 ± 0.71	
ESLD(d)	25 (24.5)	3.08 ± 0.43		3.54 ± 0.43		2.98 ± 0.65		2.66 ± 0.60		2.96 ± 0.67	
Characteristic	*n* (%)	Total educational needs		Educational needs 1		Educational needs 2		Educational needs 3		Educational needs 4	
		Mean ± *SD*	*t/F(p)*	Mean ± *SD*	*t/F(p)*	Mean ± *SD*	*t/F(p)*	Mean ± *SD*	*t/F(p)*	Mean ± *SD*	*t/F(p)*
*Received hospice, palliative, and EOL care education*^§^											
Yes	42 (42.4)	3.08 ± 0.45	−1.40 (.166)	3.06 ± 0.48	1.43 (.156)	3.09 ± 0.52	1.21 (.229)	3.13 ± 0.49	1.69 (.094)	3.05 ± 0.53	0.76 (.449)
No	57 (57.6)	2.94 ± 0.48		2.91 ± 0.50		2.94 ± 0.63		2.95 ± 0.58		2.97 ± 0.52	
*Main disease group cared for*^‡^											
CHF (a)	26 (26.0)	2.88 ± 0.58	3.40 (.021)(b > d)	2.89 ± 0.54	3.29 (.024) (b > d)	2.86 ± 0.70	2.04 (.114)	2.88 ± 0.65	2.41 (.072)	2.88 ± 0.63	2.84 (.042) (b > d)
Stroke(b)	26 (26.0)	3.19 ± 0.40		3.13 ± 0.41		3.22 ± 0.60		3.25 ± 0.53		3.21 ± 0.44	
ESRD(c)	23 (23.0)	3.05 ± 0.34		3.09 ± 0.38		3.00 ± 0.37		3.04 ± 0.46		3.06 ± 0.43	
ESLD(d)	25 (25.0)	2.82 ± 0.49		2.72 ± 0.66		2.88 ± 0.59		2.94 ± 0.45		2.80 ± 0.61	

*SD* standard deviation, *ICU* intensive care unit, *EOL* end-of-life, *CHF* congestive chronic heart failure, *ESRD* end-stage renal disease, *ESLD* end-stage liver disease

Confidence 1: pain and symptom management, Confidence 2: counselling, Confidence 3: management, Confidence 4: program. Educational needs 1: pain and symptom management, Educational needs 2: counselling, Educational needs 3: management, Educational needs 4: program

^†^*n* = 101, ^‡^*n* = 100, ^§^*n* = 99. Post Hoc: Tukey HSD

Among the four subdomains, ‘management’ showed the biggest gap between participants’ confidence and educational needs followed by ‘program’ and ‘counselling’. Confidence concerning ‘pain and symptom management’ was higher than the educational need for it.

### Correlation between total clinical experience, total knowledge, attitude, confidence, and educational needs

Total knowledge was significantly correlated with attitude (*r* = .29, *p* = .003). Total confidence was significantly correlated with total educational needs (*r* = −.21, *p* = .037) but not significantly correlated with total clinical experience (*r* = .16, *p* = .107), total knowledge (*r* = .19, *p* = .061), or total attitude (*r* = .05, *p* = .651).

### Factors affecting confidence

To identify the factors affecting nurses’ confidence in providing palliative care, three variables statistically associated with total confidence (received hospice, palliative, or EOL care education (*t* = − 2.51, *p* = .014), main disease group cared for (*F* = 5.82, *p* = .001), and total educational needs (*r* = −.21, *p* = .037) were entered into the multiple regression model. The regression model with five factors explained 18.2% of the variance in total palliative care confidence.

Two factors, ‘received hospice, palliative, or EOL care education’ and ‘disease group cared for was ESLD’, significantly affected nurses’ total confidence in providing palliative care. Participants who had received hospice, palliative, or EOL care education displayed higher confidence levels than participants without such education (std. β =0.25, *p* = .010). Further, participants who were mainly caring for patients with ESLD displayed higher confidence levels than participants who were mainly caring for patients with stroke (std. β =0.39, *p* = .001) (Table 5).

**Table 5:** Factors Affecting Confidence (*N* = 102)

Independent variables	β	*S.E.*	Std. β	*t(p)*	*R*^*2*^	Adj *R*^*2*^	*F(p)*
Constant	2.85	0.40		7.07 (< .001)	.224	.182	5.37 (< .001)
Received hospice, palliative, and EOL care education	0.30	0.11	0.25	2.63 (.010)			
Total educational needs	−0.17	0.12	−0.14	−1.40 (.164)			
Main disease group: CHF^†^	0.20	0.16	0.15	1.27 (.206)			
Main disease group: ESRD^†^	0.09	0.16	0.07	0.61 (.545)			
Main disease group: ESLD^†^	0.55	0.16	0.39	3.45 (.001)			

Tolerance: .624–.926, VIF: 1.080–1.602. ^†^Baseline (main disease group: Stroke)

*CHF* congestive heart failure, *ESRD* end-stage renal disease, *ESLD* end-stage liver disease

## Discussion

This study examined the palliative care knowledge, attitude, confidence, and educational needs in nurses who care for non-cancer patients to determine what factors affect their palliative care confidence. The results showed that the nurses’ palliative care knowledge was consistent with the results of previous studies, but lower than nurses caring for cancer patients. In addition, this study showed moderate attitudes towards palliative care for non-cancer patients. Nurses reported educational needs for management, program, and counselling services; however, when they had received education in hospice, palliative, or EOL care, they displayed significantly higher confidence than their counterparts without such education.

Nurse participants’ knowledge level was similar to that found in other studies conducted in Korea and Saudi Arabia. In 2012, all 368 Korean nurses working in cancer units, general wards, and ICUs in a tertiary hospital completed the PCQN questionnaire and obtained a mean score of 8.95 ± 2.34 [26]. In another study 365 medical and surgical ward nurses from two hospitals in Saudi Arabia obtained a mean score of 8.88 ± 1.75 on the PCQN [38]. Other studies showed that the palliative care knowledge level among nurses caring for non-cancer patients was lower than in nurses caring for cancer patients [26, 27]. Choi and colleagues reported that nurses working in cancer units had higher scores than those working in general wards or ICUs [26], and nurses’ palliative care knowledge level was significantly higher in cancer centres than in community hospitals, although the measures used to assess knowledge levels differed [27]. Palliative care was first developed for patients with end-stage cancer, and has been widely provided for patients with cancer and their family members [39]. However, many nurses and doctors caring for non-cancer patients feel uncomfortable providing palliative care because they lack the necessary education and experience [40, 41]. Furthermore, diseases progress differently, and individual patients consequently have diverse care needs [14]. For example, patients with cancer usually face steady progression and a short-term terminal phase; while patients with chronic diseases, such as organ failure, gradually decline with intermittent deterioration and recovery episodes [14]. Moreover, lack of palliative care experience could be associated with lower palliative care knowledge levels for non-cancer patients because there is not yet an integrated concept regarding the appropriate moment for transition from acute or critical care to palliative care in non-cancer patients [42]. Hence, doctors and nurses caring for non-cancer patients should be trained to understand the diverse needs and develop competency in providing timely palliative care [42]. Further research on developing education programs for nurses caring for non-cancer patients should be conducted.

In this study, nurses were highly confident in pain and symptom management. Comprehensive care of physical symptoms including pain is a principle of palliative care [43]. Nurses are essential for palliative care [20, 44] because pain and symptom management are important fundamentals in nursing. However, the participants, in this study were least confident in management and thus had a high need for management education. A previous study of 156 Korean nurses reported results consistent with this study [37]. Another study examined EOL care confidence among various types of healthcare professionals revealed that participants felt most confident in ‘providing emotional support for patients and families’, while they felt least confident in ‘providing continuity of care’ [28]. Our results indicate that educational programs for palliative care should focus on human and material resource management, systematic and ethical programs to protect patients’ and families’ rights, and counselling, including spiritual care. Palliative care management includes managing employees’, hospice teams’, and volunteers’ stress [45]. It is possible that the confidence level for management was lower in this study because most participants were staff nurses, with fewer than one-quarter charge nurses or head nurses. However, palliative care management should be strengthened and supervisors should be trained in palliative care because ‘nurses’ difficulty coping’, ‘lack of experience and education’, ‘staffing levels’ and ‘environmental circumstances’ were reported by ICU nurses to be barriers to providing terminal care [45]. Therefore, further studies should aim to identify nurses’ specific educational needs depending on their roles and professional disciplines, because differences in EOL care confidence were found among various disciplines [28]. Moreover, since many participants still incorrectly answered pain and symptom management questions, this domain should not be overlooked in the education program. Discussion about the philosophy of palliative care should also be included in education programs [46], because participants in the current study lacked this knowledge.

In this study, nurses who had received hospice, palliative, or EOL care education had higher palliative knowledge levels (although non-significant), and knowledge was positively correlated with attitude toward palliative care. A past study revealed that nurses who had completed the European Certificate in Essential Palliative Care had consistently higher PCQN scores than nurses who had only attended information sessions in their unit. In addition, their attitudes toward palliative care became more positive as their knowledge level increased [46]. Since ‘received hospice, palliative, or EOL care education’ significantly affected nurses’ total confidence in providing palliative care, and knowledge and attitude predict behaviour [47], both factors should be considered when developing educational programs to improve nurses’ competency in providing palliative care. Achora and Labrague reviewed 26 studies (published between 2000 and 2017) related to nurses’ knowledge and attitude toward palliative care and found they were affected by education and clinical experience [19]. Further, an integrative review on improving palliative care strategies reported that 14 of 68 relevant studies used educational strategies between 2000 and 2011, with 12 studies reporting improvement in palliative care [48]. Harden and colleagues asserted that ‘which contents should be included’ and ‘how to deliver the information’ could be major considerations for palliative care education [49]. In addition, Achora and Labrague suggested that palliative care should be included in nursing school curricula and palliative care education should be reinforced in clinical practice [19]. Given that hospice, palliative, or EOL care education was a significant factor in palliative care confidence, further research should be conducted to determine the ideal content and optimal delivery methods for palliative care education programs.

Knowledge level, attitude, confidence, and educational needs differed between the disease groups that participants cared for in this study. In particular, nurses caring for ESLD patients had significantly higher total confidence in providing palliative care, and participants who cared for stroke patients reported lower knowledge levels, attitude, and confidence than participants caring for ESLD patients. Consistent with this, participants caring for stroke patients reported higher palliative care educational needs than participants caring for ESLD patients. A previous qualitative study of health professionals in UK stroke units reported that palliative care was important for stroke patients and families, but there was uncertainty regarding initial transition to palliative care and integrating acute care with palliative care [50]. A 2007 study reported a lack of data for distinguishing between palliative care for stroke patients who die in the acute phase and those who die later [51]. Therefore, reliable assessments for palliative care needs in stroke patients are still needed. Further studies should examine the initial transition phase to palliative care and identify how palliative care can be provided in different ways for different disease groups.

Although this study included only nurses, managing the uncertainty of palliative care in non-cancer patients and systemizing palliative care will require cooperation among professionals. In addition, many more studies are needed to develop interdisciplinary palliative care models for non-cancer patients and their families [3].

This study had some limitations. First, the results cannot be generalised to all nurses who are caring for non-cancer patients because this study used convenience sampling methods and data were collected from only one tertiary hospital. In addition, the number of participants was low at 102, data collection was conducted in both general wards and ICUs, and this study did not include the nurses caring for chronic diseases that may also require palliative care, such as chronic obstructive pulmonary disease, dementia, and so on. Therefore, the results of this study may be limited and biased. Further large-scaled studies are needed to better generalise the results. Also, the instrument used to measure confidence and educational needs did not have reported validity, although it was reliable. Consequently, the items may not have fully assessed nurses’ palliative care confidence and educational needs. Therefore, this instrument should be validated, or a more valid and reliable instrument should be developed for future studies.

## Conclusion

Despite these limitations, this study provided solid information that can be utilised to develop palliative care educational programs. In this study, nurses who cared for patients with CHF, stroke, ESRD, and ESLD had lower palliative care knowledge and were less confident about palliative care management, programs, and counselling than they were about pain and symptom management. In addition, this study revealed that a significant factor affecting nurses’ palliative care confidence was a previous education course in hospice, palliative, or EOL care. In conclusion, to provide high-quality palliative care for non-cancer patients and their families, continuous and integrated palliative care education programs should be developed based on the nurses’ palliative care knowledge, attitude, confidence, and educational needs. Furthermore, palliative care should be specialised based on disease characteristics and coordinated professional disciplines. Future studies should be considered to explore palliative care experiences in different types of health care professionals and different types of non-cancer patients, and to develop effective training programs for palliative care specialists caring for non-cancer patients.

## Data Availability

Not applicable.

## Abbreviations

EOL: End-of-life
ELNEC: End of Life Nursing Education Consortium
ICUs: Intensive care units
CHF: Congestive heart failure
ESRD: End-stage renal disease
ESLD: End-stage liver disease
PCQN: Palliative Care Quiz for Nursing
